# Endolysosomal Mesoporous Silica Nanoparticle Trafficking along Microtubular Highways

**DOI:** 10.3390/pharmaceutics14010056

**Published:** 2021-12-27

**Authors:** Achraf Noureddine, Michael L. Paffett, Stefan Franco, Alfonso E. Chan, Sandeep Pallikkuth, Keith Lidke, Rita E. Serda

**Affiliations:** 1 Department of Chemical and Biological Engineering, University of New Mexico, Albuquerque, NM 87131, USA; anoureddine@unm.edu; 2 Fluorescence Microscopy Shared Resource, University of New Mexico Comprehensive Cancer Center, Albuquerque, NM 87131, USA; MPaffett@salud.unm.edu; 3 Internal Medicine, University of New Mexico Health Science Center, Albuquerque, NM 87131, USA; stefanfranco@yahoo.com (S.F.); ricochan65@gmail.com (A.E.C.); 4 Physics and Astronomy, University of New Mexico, Albuquerque, NM 87131, USA; pallikkuth@unm.edu (S.P.); klidke@unm.edu (K.L.)

**Keywords:** cytokinesis, microtubules, super-resolution microscopy, scanning electron microscopy, tunneling nanotube, mitotic nanotube, midbody, Flemming body, mesoporous silica nanoparticle, hyperploid cell, mitosis

## Abstract

This study examines intra- and intercellular trafficking of mesoporous silica nanoparticles along microtubular highways, with an emphasis on intercellular bridges connecting interphase and telophase cells. The study of nanoparticle trafficking within and between cells during all phases of the cell cycle is relevant to payload destination and dilution, and impacts delivery of therapeutic or diagnostic agents. Super-resolution stochastic optical reconstruction and sub-airy unit image acquisition, the latter combined with Huygens deconvolution microscopy, enable single nanoparticle and microtubule resolution. Combined structural and functional data provide enhanced details on biological processes, with an example of mitotic inheritance during cancer cell trivision.

## 1. Introduction

Cellular microtubule highways shuttle cargo, including organelles, protein complexes, and nucleic acids within cells, facilitating cell activity, shape, and mitosis [1,2]. During interphase, these highways form radial arrays of crisscrossed networks across the cell, originating at the centrosome [a.k.a. microtubule organizing center (MTOC)] near the cell nucleus. In addition to these radial highways, roadways directed towards the leading edge of motile cells originate from the Golgi [3]. Motor proteins bind to these microtubule filaments and use ATP to move cargo, including organelles, along the highways [4,5]. The motor proteins tether endocytic membranes to actin and microtubule cytoskeletons, directing the spatial organization of these organelles [6,7]. During mitosis, microtubule highways insure orderly partitioning of cellular material into the nascent daughter cells [8].

This study monitors cellular trafficking of internalized mesoporous silica nanoparticles (MSN) during various phases of the cell cycle. MSN are inorganic nanoparticles, chosen for study based on their tunable pore and particle size, providing a high drug loading capacity, biocompatibility, and promise as drug delivery or immunotherapy vehicles [9,10]. Well-characterized HeLa cells, via dynamin-2-mediated caveolar endocytosis, robustly internalize cationic MSN [11]. Endocytosis of silica nanoparticles and silicon microparticles occurs through invagination or outward movement of the cell membrane, respectively, with both resulting in the formation of membrane-bound vesicles [10,12,13,14]. Four hours post internalization of MSN by HeLa cells, vesicles housing the MSN are predominately positive for the late endosome/lysosome markers LAMP1 and LysoTracker [11]. It is well established that endosomes actively move along microtubules [6], with the majority of long movements involving dynein-driven translocation towards the cell center [15]. Sorting of endocytic material for recycling or degradation is controlled in part by spatial location, with movement towards the cell center facilitating degradation [16]. 

Similar to other cellular events, vesicular localization within cells is dynamic, and perinuclear endolysosomes retain the potential to move towards the cell periphery in the presence of appropriate triggers, such as cytoplasmic acidification or nutrient levels [17,18,19]. Another destination for cellular organelles/vesicles is transport between interphase cells through tunneling nanotubes [9,20]. These open membrane channels enable direct transfer of cytoplasmic components between adjacent cells [21]. It has been reported that tunneling nanotube diameters range from 50 to 1500 nm with lengths anywhere from a few to over 100 µm [22,23]. A discussion of international experts in basic biology concluded that tunneling nanotubes are open-ended tubular connections with variable diameters from 50 to 800 nm, able to reach lengths of 300 µm. The group also concluded that classification requires the ability for intercellular exchange of material [23].

During mitosis, cell rounding occurs, and endosomes colocalize with microtubules, which remodel to form a mitotic spindle that serves as a track for accurate segregation of cellular material [8,24]. During anaphase, endosomal compartments cluster, but remain distinct [25,26]. This is facilitated by inhibition of endosomal fusion at the onset of mitosis by regulators, such as Polo-like kinase 1 (Plk1) [25,27]. Cytokinesis, the physical separation of the dividing cell, involves ingression of a cleavage furrow midway between the two spindle poles, resulting in cytoplasm partitioning into two domains. As cytokinesis progresses, a narrow cytoplasmic mitotic bridge containing anti-parallel microtubule bundles develops, connecting the two nascent daughter cells. The final step of cell division, termed abscission, involves localized breakdown of the cytoskeleton within a region of the mitotic bridge, followed by cell separation [28]. 

This study examines cellular localization of MSN-laden endosomes at various stages of the cell cycle in order to appreciate the impact of cellular events on nanoparticle destination and dilution, both of which impact the functionality of therapeutic payloads. High-resolution microscopy, combined with electron and correlative microscopy, is used to visualize microtubular networks, both within cell bodies and cellular bridges. Cellular bridges include tunneling nanotubes and mitotic bridges. 

## 2. Materials and Methods

### 2.1. Materials 

Absolute (100%) and 95% ethanol (EtOH) were obtained from Decon Labs. Hydrochloric acid (HCl, 37%), ammonium nitrate (NH_4_NO_3_), cetyltrimethylammonium chloride (CTAC; 25 wt.% in H_2_O), cyclohexane, tetraethyl orthosilicate (TEOS), triethanolamine (TEOA), and (3-aminopropyl)triethoxysilane (APTES) were purchased from Millipore Sigma (Burlington, MA, USA). DyLight fluorophores, LysoTracker^®^ Red DND-99, Alexa Fluor 647 phalloidin, and Prolong Gold with DAPI were purchased from Thermo Fisher Scientific (Grand Island, NY, USA). 

### 2.2. Methods

#### 2.2.1. Cell Culture

Human HeLa cervical and A549 lung cancer cells were cultured at 37 °C in 5% CO_2_ in Eagle’s modified essential media (EMEM) or F-12 media, respectively, supplemented with 10% fetal bovine serum and 10 mL/L 10,000 units penicillin/10 mg streptomycin/mL. Cells and media were purchased from ATCC (Manassas, VA, USA). HeLa cells were chosen based on their abundant use in mitotic studies and the presence of intercellular bridges [9]. A549 were selected to compare temporal MSN uptake by a different cancer type.

#### 2.2.2. Synthesis of Primary Amine-Bearing Monodisperse MSN (MSN-NH_2_)

In a 100 mL round bottom flask, 0.18 g (1.8 mmol) TEOA, 24 mL (72.6 mmol) CTAC and 36 mL of distilled water were stirred at 400 rpm and heated for 1 h to bring the solution to 50 °C. Next, 20 mL of a solution of TEOS in cyclohexane (20% *v/v*) was added to form the biphasic system. The reaction was kept at 50 °C under with stirring for 16 h. A solution of APTES (220 µL) in ethanol (400 µL) was then added to the aqueous phase and the reaction continued for 4 h. Next, the top organic phase was removed and the bottom aqueous phase containing the nanoparticle suspension was centrifuged. The isolated pellet was washed in pure ethanol twice using successive sonication and centrifugation steps. The removal of CTAC was achieved by washing the suspended particles in NH_4_NO_3_ (6 g L^−1^) in ethanol, followed by 1% HCl in ethanol. Each suspension was sonicated for 15 min and centrifuged. Each cycle of centrifugation was performed at 50,000 relative centrifugal force (rcf) for 15 min at room temperature. For MSN-NH_2_-Cy3, the same procedure was followed but following the addition of TEOS a solution containing 3 µL APTES and 3 µg Cyanine3 (Cy3) NHS ester in 500 µL ethanol was added with stirring at 22 °C for 2 h.

#### 2.2.3. Conjugation of MSN-NH_2_ with DyLight Fluorophores

A suspension of surfactant-free MSN-NH_2_ (5 mg) in ethanol (1 mL) was sonicated with an ethanolic solution of 3 mg/mL DyLight™ fluorophore (594 or 633; Thermo Fisher Scientific) and kept under gentle stirring overnight at room temperature. Next, the suspension was centrifuged (20,000 rcf) and washed with pure ethanol until the supernatant became clear (approximately 3 times). The labeled MSN were stored in ethanol at 4 °C in the dark.

#### 2.2.4. Nanoparticle Characterization 

Transmission electron microscopy (TEM) images were acquired using a JEOL 2010 (JEOL, Tokyo, Japan) instrument equipped with an Orius digital camera system (Gatan, Warrendale, PA, USA) at 200 kV. Nitrogen adsorption–desorption isotherms of MSN were obtained using a Micromeritics ASAP 2020 (Micromeritics Instruments Corp., Norcross, GA, USA) at 77 K. Samples were degassed at 60 °C for 12 h before measurements. The surface area was calculated following the Brunauer–Emmett–Teller (BET) equation and the pore size was obtained using DFT theory and standard Barrett–Joyner–Halenda (BJH) methods from adsorption branch.

Hydrodynamic size and zeta potential data were acquired using a Malvern Zetasizer Nano-ZS equipped with a He–Ne laser (633 nm) and non-invasive backscatter optics (NIBS) (all components were purchased from Malvern Panalytical, Inc., Westborough, MA, USA). All samples for DLS measurements were suspended in distilled water at a 1 mg/mL concentration. Samples were washed 3 times through centrifugation prior to measurements. Measurements were acquired at 25 °C. DLS measurements for each sample were obtained in triplicate and then the Z-average diameter (by intensity) was used for all reported hydrodynamic size values. The zeta potential for all the samples was also measured in distilled water in triplicate according to Smoluchowski theory. All reported values correspond to the average of 3 independent measurements of at least 20 accumulated runs.

#### 2.2.5. Flow Cytometry

Cells were seeded into 6-well plates at a density of 1 × 10^5^ cells per well. After 24 h, 10 µg/mL fluorescent MSN were added for the indicated durations for uptake and transfer studies. For transfer studies, cells containing unique fluorophore-labeled MSN were combined for an additional 24 h. Cells were analyzed using a BD™ LSRII, LSRFortessa flow cytometer (BD Biosciences, San Jose, CA, USA) using FACSDiva software (BD Biosciences, San Jose, CA, USA). 

#### 2.2.6. Huygens Super-Resolution Confocal Microscopy 

HeLa cells were seeded onto glass cover slips in 6-well plates at a density of 1 × 10^5^ cells per well. After 24 h, fluorescent MSN were added as needed in fresh complete media at 10 µg/mL for the indicated amount of time. When required, LysoTracker™ Red DND-99 (75 nM; Thermo Fisher, Carlsbad, CA, USA) was added to the cells in pre-warmed culture media during the final 30 min of incubation as described in the manufacturer’s protocol. After the final incubation, cells were washed with PBS, fixed with 4% paraformaldehyde in PBS for 15 min with pre-warmed solutions followed by overnight refrigeration, rinsed twice with PBS, and permeabilized with 0.1% Triton-X in PBS for 15 min. Cells were then blocked with 1% BSA for 20 min and then labeled with 5 units/0.5 mL Alexa Fluor 647 (AF647) phalloidin (Thermo Fisher) and/or 10 µg/mL mouse anti-α-tubulin antibody-Alexa Fluor 488 (Thermo Fisher, clone B-5-1-2) in 1% BSA for 1 h. After washing with PBS, slides were mounted using Prolong Gold with DAPI (Thermo Fisher). Confocal images were acquired with a 63×/1.4 NA oil objective in sequential scanning mode, using a sub-Airy pinhole size of 0.6 AU, using a TCS SP8 confocal system microscope (Leica Microsystems, Wetzlar, Germany) equipped with tunable and white light and 405 nm diode lasers, and metal halide fluorescence and halogen transmission lamps (Leica Microsystems, Wetzlar, Germany). Detectors included two hybrid spectral detectors (HyD), two spectral single molecule HyDs, and standard spectral, and transmitted PMTs. The system was supported with LASX and Huygens Essential B.V. software by Scientific Volume Imaging (Hilversum, The Netherlands). Microscopy files were exported as 8-bit TIFF (merged fluorophore) or JPEG (single fluorophore) images and figures were assembled using either Illustrator or Photoshop (both from Adobe Inc., San Jose, CA, USA), with gamma levels adjusted as needed to enhance contrast and brightness. 

#### 2.2.7. Stochastic Optical Reconstruction Microscopy (dSTORM)

Cells were cultured on 25 mm glass coverslips for 18 h followed by incubation with 10 µg/mL DyLight 633-labeled MSN for 5 h at 37 °C. After two warm PBS washes, cells were incubated in warm 4% paraformaldehyde for 15 min, followed by 18 h at 4 °C. Cells were washed in PBS and the coverslip was mounted in an in-house manufactured titanium holder. Imaging (TNG) buffer, consisting of an enzymatic oxygen scavenging system and primary thiol [50 mM Tris, 10 mM NaCl, 10% *w/v* glucose, 168.8 U/mL glucose oxidase (Sigma #G2133), 1404 U/mL catalase (Sigma #C9332), and 32 mM 2-aminoethanethiol (MEA), pH 8] was added (1.5 mL, made fresh) and a coverslip was set on top to prevent oxygen exchange, with care to eliminate bubbles that would deflect light. Five locations were selected and 40,000 events collected from each site using an imaging system custom built from an IX71 inverted microscope (Olympus Life Sciences, Tokyo, Japan) equipped with an Olympus APO N 60×/1.49 oil immersion lens (Olympus Life Sciences, Tokyo, Japan) at 36 mW laser power at 647 nm, as measured just before the objective. Cells were bleached by collecting 20,000 events per location in PBS with the power intensity increased 10-fold, followed by 10 min incubation in sodium borohydride (1 mg/mL PBS; made immediately before use). Cells were incubated with rabbit anti-α-tubulin antibody-Alexa Fluor 647 (EP1332Y; Abcam, Cambridge, MA, USA) at 10 μg/mL in PBS containing 1% bovine serum albumin for 30 min at room temperature. After 2 PBS washes, TNG buffer was added and images reimaged. This process was repeated using 10 μg/mL mouse anti-human alpha tubulin AF647 (Sigma-Aldrich, St. Louis, MO, USA; clone T6074).

#### 2.2.8. Scanning Electron Microscopy (SEM) Imaging of Cells 

HeLa or A549 cells were seeded in 24 well plates containing 5 × 7 mm silicon chip specimen supports (Ted Pella, Inc., Redding, CA, USA) at 5 × 10^4^ cells per well. Cells were then incubated with 10 µg/mL MSN for 24 h, and then processed for SEM imaging as previously described [13]. Secondary electron images were acquired under high vacuum, at 20 kV with a spot size of 5, using an FEI Quanta 3D FEG, (FEI, Hillsboro, OR, USA). Alternatively, images were acquired using a Sigma SEM (Carl Zeiss Microscopy, Göttingen, Germany) at 1 kV. Prior to imaging, dehydrated samples were sputter-coated with approximately 5 nm gold-palladium using a ACE600 coater (Leica Microsystems, Wetzlar, Germany). Select images have been pseudo-colored using Adobe Photoshop (Adobe Systems Incorporated, San Jose, CA, USA) and gamma levels adjusted to enhance image contrast and brightness.

#### 2.2.9. Correlative Microscopy

HeLa cells were seeded onto 22 × 22 × 0.17 mm indium tin oxide coated Zeiss cover slips containing 3 reference markers in 6-well plates at a density of 0.75 × 10^5^ cells per well. After 24 h, 10 µg/mL fluorescent MSN were added for an additional 24 h and cells were labeled for imaging as previously described. Cover slips were mounted on glass slides using mounting media and dental molding paste.

Confocal images were acquired using the LSM 800 Airyscan Confocal Microscope equipped with four solid-state lasers, GaAsp PMT detectors, Zen Blue Image Acquisition, and Zen Shuttle and Find software (all components were purchased from Carl Zeiss Microscopy, Göttingen, Germany). Following image acquisition, the coverslip was gentle removed from the slide, washed repeatedly with PBS, dehydrated as previously described and mounted on an SEM stub using double-sided carbon tape. Samples were sputter coated with 5 nm gold-palladium, and SEM images were acquired and merge with fluorescent images using a Zeiss Sigma SEM equipped with Zen Shuttle and Find software.

## 3. Results

### 3.1. Characterization of Mesoporous Silica Nanoparticles (MSN)

**Figure 1:**
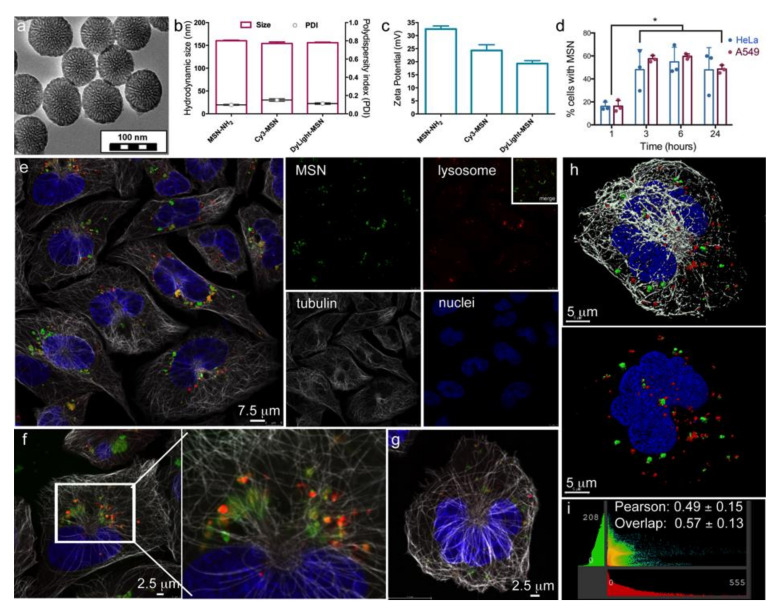
Endolysosomal localization of internalized MSN. (**a**) Transmission electron micrograph of MSN showing size and porous structure. (**b**) Dynamic light scattering analysis of hydrodynamic size and polydispersity (in water). (**c**) Zeta potential measurements before and after fluorophore grafting. (**d**) Flow cytometry analysis of MSN internalization from 1 to 24 h in HeLa or A549 cells. Merged (**e**–**h**) or single (**e**) fluorophore confocal micrographs of LysoTracker™ Red (red), AF488 anti-α-tubulin antibody (white), and DAPI (blue) labeled HeLa cells following 24 h incubation with DyLight™ 633-labeled MSN (green). The 2D (**f**) and 3D (**g**) projection images, with surface rendering in (**h**). (**i**) Representative MSN and lysosome colocalization graph and mean Pearson’s correlation coefficient and overlap. * *p* < 0.05.

Based on easy surface functionalization, monodispersity, homogeneous size, slow degradation rate, and their potential use in drug delivery or immune therapy, MSN were chosen to monitor nanoparticle trafficking in cells using HeLa cervical cancer cells as a model cell line. Surface modification of the MSN is illustrated in Supplemental Figure S1a. Transmission electron micrographs support monosized spherical MSN with a dendritic pore structure (Figure 1a). The very thin dark layer observed on the outer surface is indicative of the condensation of (3-aminopropyl)triethoxysilane (APTES) added in situ on the preformed MSN. The hysteresis loop in the nitrogen sorption isotherm between P/P° = 0.4 and 0.7 is a clear indication of the mesoporosity of the material (Supplemental Figure S1b). The size of the pores was calculated from the adsorption branch with Barrett–Joyner–Halenda theory and shows a pore size distribution centered at 5 nm. The Brunauer–Emmett–Teller surface area was calculated to be around 416 m^2^/g with a total pore volume near 1.2 cm^3^/g, indicating highly accessible pores. Further, colloidal stability of MSN-NH_2_ was demonstrated using dynamic light scattering, with the hydrodynamic size of particles being around 160 nm in diameter before and after dye grafting (Figure 1b). The polydispersity index (PDI) was less than 0.2, supporting high size homogeneity (Figure 1b). The zeta potential showed that surface charge of MSN-NH_2_ (+32 mV) decreased after dye grafting (+17 to +24 mV), supporting that surface amines reacted with the NHS ester groups of the dyes (Figure 1c).

### 3.2. Intracellular Localization of MSN

MSN are internalized through endocytosis, leading to localization in membrane-bound vesicles [9]. Using flow cytometry, internalization of fluorescent, cationic MSN by both HeLa and A549 human cancer cells was demonstrated, with a plateau in uptake beginning at 3 h (Figure 1d). At 24 h post addition of MSN to HeLa cells, most MSN were located in the perinuclear region of the cell, with some MSN colocalizing with the acidic late endosome and lysosome marker LysoTracker™ Red (Figure 1e–h). Huygens Essential software was used to evaluate co-localization of MSN with LysoTracker™ Red using Gaussian minimum for threshold setting (Figure 1i). The resulting Pearson correlation of 0.49 ± 0.15 and overlap of 0.57 ± 0.13 indicate that approximately 50% of MSN were located in acidic late endosomes/lysosomes 24 h post addition to HeLa cells. Based on these data and current literature, MSN are assumed to exist in endo-lysosomes.

### 3.3. Microtubule Trafficking of MSN-Endolysosomes

**Figure 2:**
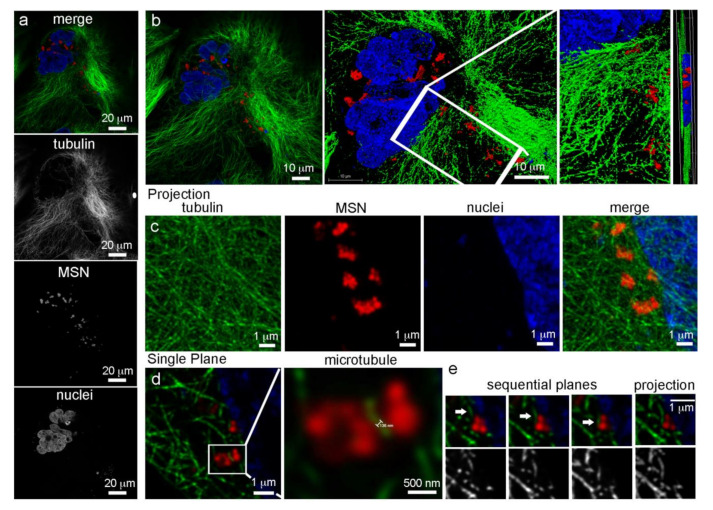
Microtubule highways in HeLa cells. (**a**,**b**) Single plane and 3D confocal micrographs showing individual fluorophore and merged images of a HeLa cell incubated with MSN for 24 h [microtubules in green (AF488), nuclei in blue (DAPI), and MSN in red (DyLight 633)]. (**c**) High magnification single and merged fluorophore projection images showing perinuclear MSN-laden endosomes and their association with microtubules. (**d**,**e**) Single plane and projection deconvoluted micrographs showing MSN association with single microtubule filaments. The size bar in “d” shows resolution at 136 nm.

**Figure 3:**
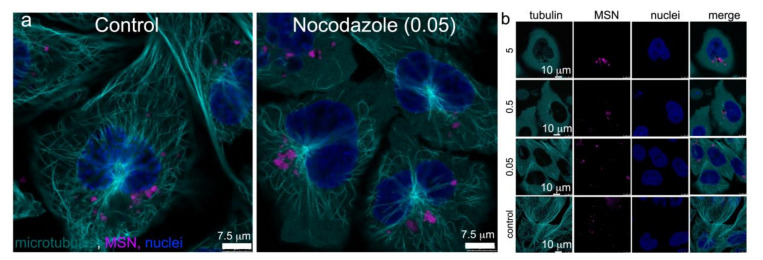
Disruption of MSN-endolysosome trafficking through inhibition of tubulin polymerization. (**a**,**b**) Confocal fluorescent micrographs of HeLa cells incubated with DyLight 633 MSN (magenta) in the absence (control) or presence of nocodazole. Cells were post fixation-labeled with AF488 anti-α-tubulin antibody (cyan; microtubules) and DAPI (blue; nuclei). (**a**) Comparison of control and nocodazole-treated (0.05 µg/mL) HeLa cells. (**b**) HeLa cells incubated with 0–5 μg/mL nocodazole.

High resolution confocal microscopy using sub-Airy pinholes followed by computational image deconvolution [29,30] was used in combination with three-dimensional (3D) volume imaging to visualize microtubule arrays emanating from centrosomes near the cell nucleus and their association with MSN-laden endosomes 24 h after addition of MSN to HeLa cells (Figure 2). Microtubules were visualized using Alexa Fluor 488 anti-tubulin antibody (white) and MSN were conjugated to DyLight 633 (green). Confocal z-stacks are shown in 2D (Figure 2a) and 3D (Figure 2b), with the latter also shown following surface rendering at two magnifications and cropped near the nucleus. Deconvoluted higher magnification projection images (single fluorophore or merged) are able to distinguish individual MSN and microtubules (Figure 2c). Further sequential magnification reveals a resolution of 136 nm (Figure 2d). Figure 2e shows MSN-laden endosomes associating with microtubules via sequential z-planes. Dependence of MSN-laden endosome trafficking on microtubules was supported by nocodazole inhibition of microtubule assembly/disassembly dynamics [31], resulting in clustering of MSN-loaded endosomes near remaining microtubules (Figure 3) [6]. When interphase microtubules are completely depolymerized by nocodazole, endolysosomes become randomly distributed in the cytoplasm [31,32]. 

### 3.4. Intercellular Transport of MSN via Tunneling Nanotubes

**Figure 4:**
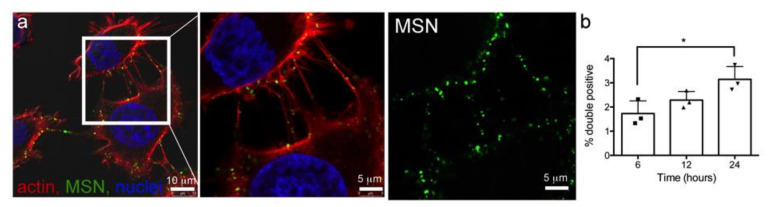
Cell-to-cell trafficking of MSN-laden endosomes via cellular bridges. (**a**) HeLa cells were incubated with DyLight 594-conjugated MSN (green) for 10 min, followed by fixation and labeling with AF647 phalloidin (red; actin) and DAPI (blue; nuclei). Merged and single fluorophore (gamma-adjusted) micrographs showing MSN in cellular bridges. (**b**) Flow cytometry analysis of double fluorescent HeLa cells 6–24 h after mixing two HeLa cell populations containing distinct single positive MSN. * *p* < 0.05.

Homotypic and heterotypic intercellular transport of MSN via tunneling nanotubes in interphase monocytes and HeLa cells was recently demonstrated [9]. Herein association of MSN with tunneling nanotubes 10 min after the addition to HeLa cells is shown (Figure 4a). MSN are located at the cell periphery and along the nanotubes. Gamma levels have been adjusted on the single fluorophore MSN image to emphasize the location of all MSN within the imaging field. While there was a significant temporal increase in cell-to-cell transfer of MSN from 6 to 24 h (1.7 vs. 3.1%, *p* = 0.03), it was detected in only a small population of cells using flow cytometry (Figure 4b). Based on low MSN transfer between cells over time, the high percentage of MSN associated with nanotubes at 10 min is likely based on trafficking of MSN along TNTs. While thick TNTs containing microtubules allow movement of vesicles within the structure, thin TNTs lacking microtubules use constitutive membrane flow to move beads and bacteria along the nanotube surface (a.k.a. surfing) [33]. Rehman et al. [34] reported that syndecans present on filopodial extensions are instrumental in processing and transporting lipoplexes to the cell body for endocytosis. It is feasible the syndecans or other glycocalyx proteoglycans facilitate movement of MSN along the TNT surface.

### 3.5. Intercellular Bridges/Connections during Mitosis 

**Figure 5:**
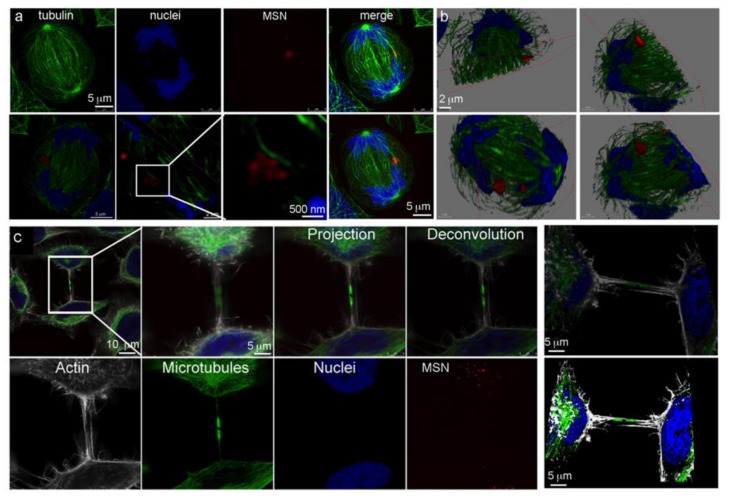
MSN-endolysosome localization during anaphase and telophase in HeLa cells. (**a**,**b**) Huygens deconvoluted fluorescent projection (**a**) and 3D (**b**) micrographs showing sister chromatid separation during anaphase (green: microtubules; anti-α-tubulin antibody-AF488; red: MSN; DyLight 594; blue: nuclei, DAPI). 3D images are displayed with and without sectioning. (**c**) Projection and 3D micrographs of a late cytokinetic HeLa cell containing internalized Cy3-MSN (red). Cells were labeled post fixation with AF647 phalloidin (white; actin), AF488 anti-α-tubulin antibody (green; microtubules) and DAPI (blue; nuclei).

**Figure 6:**
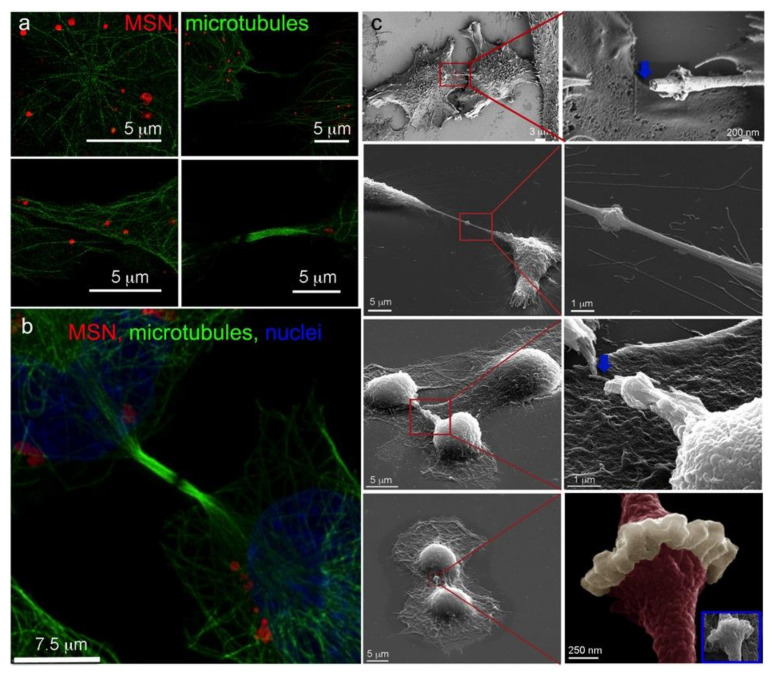
Super-resolution and topographic imaging of mitotic bridges. (**a**) Sequential super-resolution (dSTORM) labeling and imaging of HeLa cells incubated with DyLight 633-labeled MSN (red) for 5 h at 37 °C and mouse anti-human alpha tubulin AF488 (green). (**b**) HyVolution deconvoluted fluorescent confocal microscopy image of microtubule-rich (green) mitotic bridges following 24 h incubation with DyLight 633-labeled MSN (red; nuclei labeled with DAPI and shown in blue). (**c**) Scanning electron micrographs of four telophase HeLa cells with midbody regions amplified as indicated by red boxes. Arrows (blue) indicate procedural breaks in nanotubes. The final midbody image is false-colored red with the original grayscale image presented in the inset.

During early mitosis, cells round, and intercellular connections, specifically tunneling nanotubes and gap junctions, dissociate. Confocal micrographs show that MSN-laden endosomes in rounded anaphase cells cluster along polar microtubules forming the central spindle (Figure 5a,b). The majority of MSN-endolysosomes are located centrally, with a smaller cluster located near the spindle pole. The 2D confocal micrographs show that following actomyosin contraction and creation of the central furrow that separates the cytoplasm in two, MSN-endosomes are dispersed throughout the cytoplasm (Supplemental Figure S2). Following ingression of the cleavage furrow, microtubule bundles are reorganized into a mitotic bridge separating the two nascent daughter cells (Figure 5c). MSN-laden endosomes are seen dispersed juxtapose to the cell nuclei. Super resolution imaging using stochastic optical reconstruction microscopy (dSTORM; Figure 6a) and sub-Airy/deconvolution imaging (Figure 6b) show MSN-endolysosomes near the apex of the mitotic bridge, however, they are absent from within it.

### 3.6. Vesicle Trafficking and the Flemming Body

Secondary electron imaging of HeLa cells in the process of cytokinesis revealed the surface topography of the mitotic bridge. In Figure 6c, cells are shown at magnifications ranging from 2000 to 10,000× in the left column, with the boxed regions magnified at 20,000–200,000× in the right column. The final image has been pseudo-colored to further highlight the topography of the Fleming body, with the original image included as an inset. Images in the top row were acquired using a Zeiss Sigma SEM while the remaining images were acquired using a Quanta 3D FEG (FEI, Hillsboro, OR, USA). Mitotic bridges varied in length, as would be expected based on the stage of separation, with lengths shown ranging from 5 to 30 µm. Bridge diameters ranged from approximately 250 to 760 nm, with midbody rings near 1000–1200 nm (Figure 5c, Supplemental Figure S3). Thinner cellular bridges (aka mitotic nanotubes) connecting one of the mitotic cells to an interphase cell is seen in the bottom left micrograph in Supplemental Figure S3 (white circle), with a representative diameter near 100 nm. The structures of a mitotic bridge and Flemming body are also shown for a human A549 lung cancer cell in Supplemental Figure S4, with similar appearance to those presented for HeLa cells.

### 3.7. Trivision of a Hyperploid Cell

**Figure 7:**
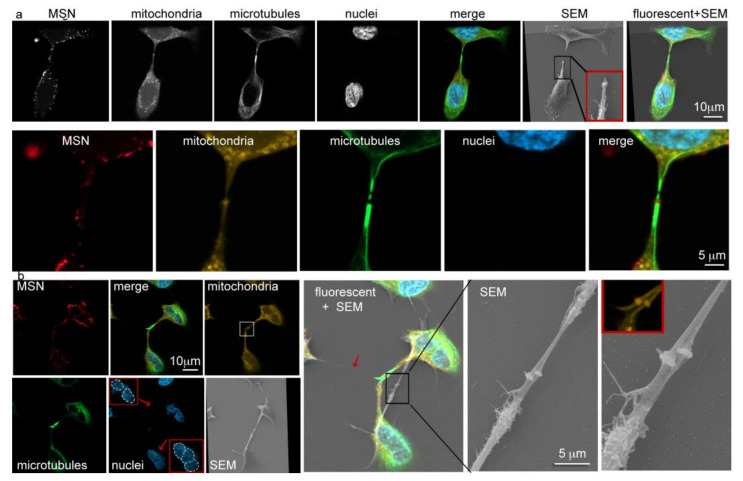
Correlative scanning electron and fluorescent microscopy imaging of a telophase HeLa cell. Merged and single fluorophore confocal and SEM images of two telophase HeLa cells [(**a**) division; (**b**) trivision] following 1 h incubation with 10 µg/mL DyLight 550-conjugated MSN (red). Cells were fixed and labeled with AF488 anti-α-tubulin antibody (microtubules; green), MitoTracker Red (mitochondria; yellow) and DAPI (nuclei; blue; red box insets show individual nuclei in (**b**)). Fluorescent and SEM images were acquired using the Zeiss Airy Scan confocal microscope and the Zeiss Sigma SEM, respectively. Shuttle and Find software was used to overlay the images. The arrow (cyan) indicates the SEM procedural break site in the mitotic bridge.

To further capture the essence of cells in the process of cytokinesis, correlative microscopy was used to integrate the ultra-structural details of electron microscopy with the functional data obtained using fluorescent microscopy. Using Shuttle and Find software (Carl Zeiss Microscopy, Göttingen, Germany) and fiducial markers, secondary electron and merged fluorescent images were integrated. A mitotic cell shown in Figure 7a in both grayscale and false-colored, displays two microtubule gaps within the mitotic bridge, one at the site of the midbody and one at the purported abscission site. A positive MitoTracker signal at the site of the midbody indicates the presence of mitochondria or a membrane potential. Unlike images presented in Figure 5 and Figure 6, MSN are present within the mitotic bridge in Figure 7a. The midbody can be seen magnified in the inset present in the SEM image. The significance of correlative microscopy is further validated in Figure 7b, where fluorescent microscopy shows three cells connected by intercellular bridges. Thick microtubule bundles present within the bridges support that they are mitotic bridges. While one daughter cell has two nuclei, another is present with three nuclei (Figure 7b; as indicated in insets). The mitotic bridge connecting two of the nascent daughter cells is broken in the electron micrograph (red arrow in merged fluorescent/SEM image in Figure 7b) as a result of the SEM dehydration or imaging process, but the presence of two midbodies, each in distinct mitotic bridges is shown. In summary, mitosis of a hyperploidy cancer cell resulted in three nascent daughter cells, with a variable number of nuclei in the progeny.

## 4. Discussion

MSN were rapidly internalized by HeLa and A549 cells, with the proportion of nanoparticle positive cells reaching a plateau at 3 h. Approximately half of the MSN in HeLa cells were located in late endosomes or lysosomes 24 h post addition. The remaining intracellular MSN were likely located in early (Rab5, EEA1) or recycling endosomes (Rab4, Rab35, Rab11) [35,36,37]. Recently the existence of both homotypic and heterotypic intercellular transfer of MSN-endolysosomes between monocytes and HeLa cells through microtubule-containing cytoplasmic bridges termed tunneling nanotubes was demonstrated [9]. Wang and Gerdes reported that healthy pheochromocytoma cells transfer mitochondria to stressed homotypic cells via microtubule-containing tunneling nanotubes, resulting in increased cell survival [38]. Wang and colleagues [39] reported that MSN impair lysosomal function in hepatocytes by damaging the organelle’s ultrastructure, increasing membrane permeability, and downregulating expression of lysosomal proteases. Most in vitro studies report a lack of MSN toxicity up to 100 µg/mL, with high concentrations (e.g., 1 mg/mL) inducing reactive oxygen species [40]. The maximum tolerated dose for fluorescent MSN in preclinical mouse studies was found to be 50 mg/kg (1 mg/mouse) [41]. Cell type (e.g., primary versus established) and nanoparticle properties (e.g., surface charge and size) have a huge impact on nanoparticle cytotoxicity, with cationic, small nanoparticles being more toxic than their neutral or anionic counterparts [42]. Intercellular exchange of MSN was rare, indicating that enhanced survival of HeLa cells via elimination of potentially compromised MSN-endolysosomes to neighboring cells was not a factor. A lack of MSN cytotoxicity was as expected based on the use of an established cell line, MSN concentration, and the presence of serum.

Shorter cytoplasmic channels connecting cells are known as gap junctions. These channels exist as dense arrays in regions where cell membranes are closely apposed (roughly 2–3 nm) with intercellular lengths from 14 to 25 nm [43]. Unlike tunneling nanotubes, which can expand to accommodate microparticles and organelles [20], passage of material through gap junctions is limited to small molecules and ions [44]. In addition to existing on the cell body, these channels are also present on the membrane of tunneling nanotubes [45]. As cells enter mitosis and rounding initiates, these gap junctional communications with surrounding cells halts, however, plasma membrane bridges, now termed mitotic nanotubes, exist to mediate communication between cells [46]. The average length of mitotic nanotubes in HeLa cells is reported to be 5.8 µm [46]. Despite a reported absence of microtubules in these nanotubes, they have been shown to have the ability to transport Rab11-positive vesicles between mitotic cells and adjacent cells. Using scanning electron microscopy, we confirmed the existence of mitotic nanotubes connecting telophase and intraphase HeLa cells. The diameter of a representative mitotic nanotube was 108 nm, with an average length consistent with the reported 6 µm.

Throughout mitosis, the distribution of endosomes is regulated by microtubules and proteins, the latter including Rab, Arf GTPases, and ESCRT proteins [47]. During prophase, centrosomes duplicate and as they separate, with endosomes splitting into two clusters that accompany each centrosome [48]. During metaphase, conflicting reports exist regarding the location of early compared to late endosomes [48]. Next, during anaphase, endosomes are transported to the cleavage furrow, facilitating cytoskeleton dynamics and accurate inheritance [8,49,50]. Consistent with this, LAMP-1 positive lysosomes have been shown to be located near mid-zone microtubules, with lesser amounts near the spindle poles [51]. In further agreement, using 3D confocal imaging of an anaphase cell, we demonstrated that the majority of MSN-endolysosomes were located in association with polar microtubules midway between the spindle poles, with a minor population associating with microtubules near the spindle pole. Following cleavage furrow ingression, MSN-endolysosomes were scattered throughout each of the nascent daughter cells.

Following ingression of the cleavage furrow and completion of actomyosin ring contraction, nascent daughter cells remain connected by a mitotic bridge. Bergeland demonstrated that Rab11 is essential for late stages of cytokinesis, possible moving endosomes into the mitotic bridge to shuttle membrane and abscission machinery [52]. Using scanning electron microscopy, select images presented within demonstrated that HeLa cell mitotic bridges varied in length depending on stage of cytokinesis, ranging from 5 to 30 µm, with diameters from 250 to 760 nm. At the center of the mitotic bridge an electron dense structure exists, termed the midbody (a.k.a. Flemming body, matrix, dark zone, ring, or stem body) [53,54,55]. High magnification electron microscopy (200,000 times) revealed the surface topography of the midbody, with a ring diameter of approximately 1000–1200 nm. dSTORM (Olympus Life Sciences, Tokyo, Japan) and sub-Airy/Huygens deconvolution high-resolution microscopy (Leica Microsystems, Wetzlar, Germany) enabled us to visualize MSN-endolysosomes localized near the mitotic bridge apex during cytokinesis. Using correlative microscopy, MSN-endolysosomes were seen (though infrequently) within the mitotic bridge. Late mitotic bridges contain specific microtubule-associated endosomes, such as those positive for FIP3 and Rab35 [56]. FIP3 is an effector protein for Rab11 that facilitates endosomal recycling and cytokinesis [56,57]. Fusion of these endosomes to the bridge prepares the cell for abscission. While MSN-laden endo-lysosomes were seen in several images of mitotic bridges, their presence was rare as expected based on the majority existing as degradative vesicles. 

During malignant transformation, some cells are rendered hyperploid, with tetraploid being the most common [58]. This contributes to genome instability through genomic constraints on mitotic machinery. Furthermore, hypertriploid HeLa cells have been shown to have a higher frequency of trivisions relative to divisions [59]. It has been hypothesized that trivisions could lead to random aneuploidy and the generation of new cancer-specific karyotypes. Our correlative microscopy data shows variable chromosomal (nuclei) inheritance following trivision of a HeLa cell supporting speculations that hyperploidy accelerates genome instability. All three daughter cells inherited MSN-laden endo-lysosomes. While normal cell division results in equal distribution of endosomes among daughter cells [8,60], the selected confocal z-slice presented in Figure 6b shows a greater number of MSN-containing vesicles in the diploid daughter cell compared to the haploid progeny. This observation, if validated across all z planes and in a large number of mitotic tetraploid cells, indicates unequal nanoparticle (and payload) delivery to daughter cells of hyperploid cells. In addition, the relevance of MSN-endolysosomes in the mitotic bridges of hyperploid cells warrants further investigation. 

This study shows an overlooked aspect of the trafficking of internalized nanoparticles by cancer cells by emphasizing how endolysosomal nanoparticles navigate throughout and between cells during the various phases of the cell cycle. While previous studies show high intercellular exchange of MSN-endolysosomes between macrophages and HeLa cells, homotypic exchange of MSN among HeLa cells was rare. Since macrophages are scavenger cells that internalize a large proportion of injected nanomaterials, this provides a mechanism for nanoparticle delivery to cancer cells, with MSN being retained within the cancer cells. While mitotic partitioning of MSN-endolysosomes was not impaired by the presence of MSN, mitotic inheritance from hyperploid cells warrants further investigation. Partitioning of MSN into daughter cells reduces the therapeutic payload, making them less effective at killing cancer cells. MSN destination within the cell is highly relevant but more important is release of the payload and payload arrival at the target site. For drugs that block mitosis, rapid intracellular release from nanocarriers inhibits cancer cell proliferation and negates dilution by mitosis.

## Data Availability

All data are included in the main manuscript and supplemental material. Raw data are available upon reasonable request.

## Supplementary Materials

The following figures are available online at https://www.mdpi.com/article/10.3390/pharmaceutics14010056/s1, Figure S1: Mesoporous silica nanoparticle (MSN) characterization, Figure S2: During late telophase-cytokinesis endosomes distribute throughout the nascent daughter cells, Figure S3: Mitotic nanotubes and sizing of mitotic bridges, Figure S4: Topographic imaging of a mitotic bridge and midbody in A549 cells.
