# Supplementary material for: Endolysosomal Mesoporous Silica Nanoparticle Trafficking along Microtubular Highways
Source: Pharmaceutics. 2021 Dec 27;14(1):56. doi: 10.3390/pharmaceutics14010056 (PMC8781846; doi:10.3390/pharmaceutics14010056)
Supplement: Supplementary file 1 [file pharmaceutics-14-00056-s001.zip › pharmaceutics-1482904-supplementary.pdf]

pharmaceutics

Supplementary Materials: Endolysosomal Mesoporous Silica Nanoparticle Trafficking along Microtubular Highways

Achraf Noureddine , Michael L. Paffett , Stefan Franco , Alfonso E. Chan , Sandeep Pallikkuth , Keith Lidke and Rita E. Serda

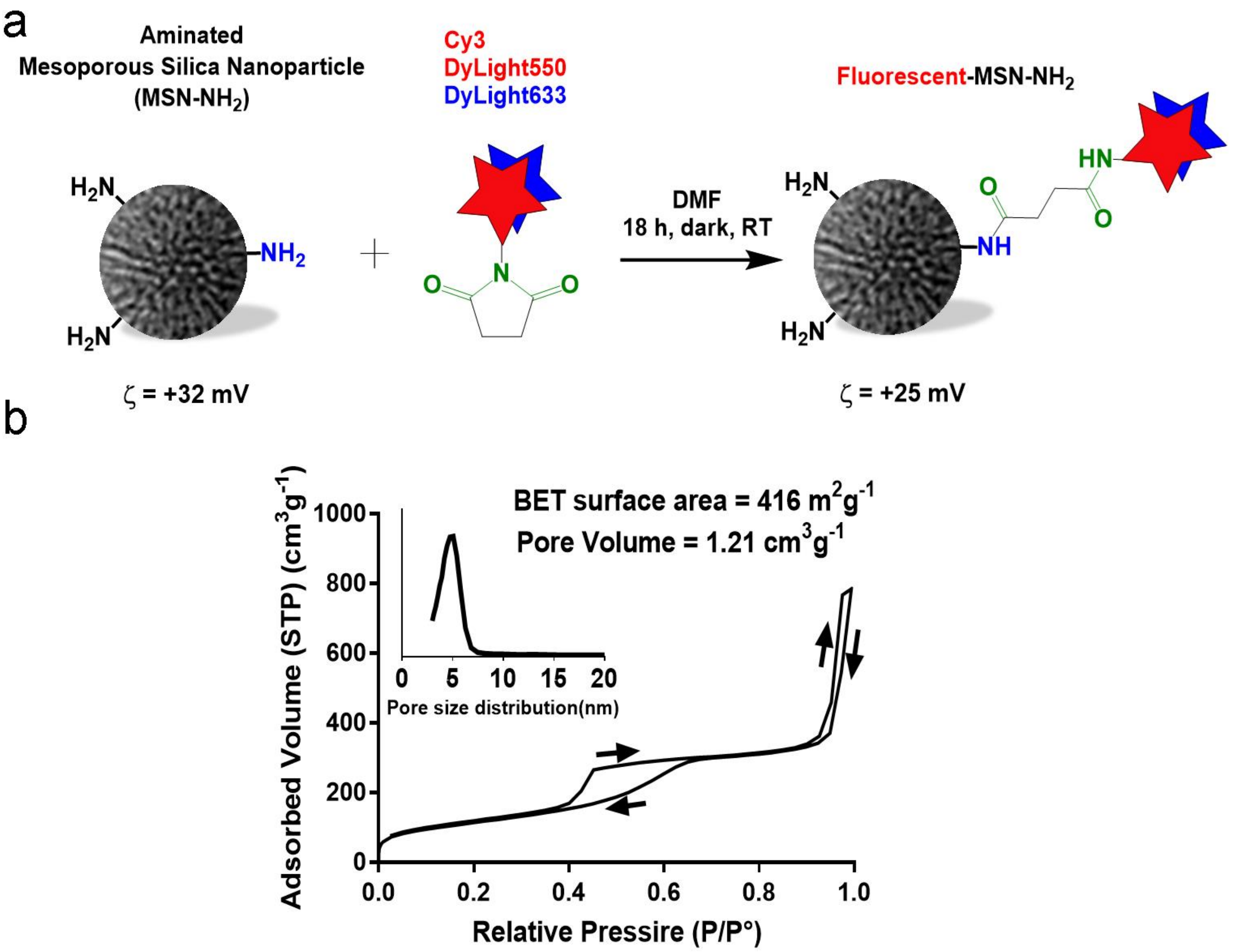

Figure S1. Mesoporous silica nanoparticle (MSN) characterization. a).sorption isotherm and pore volume distribution (inset) from the adsorption branch of the isotherm according to the BJH method.

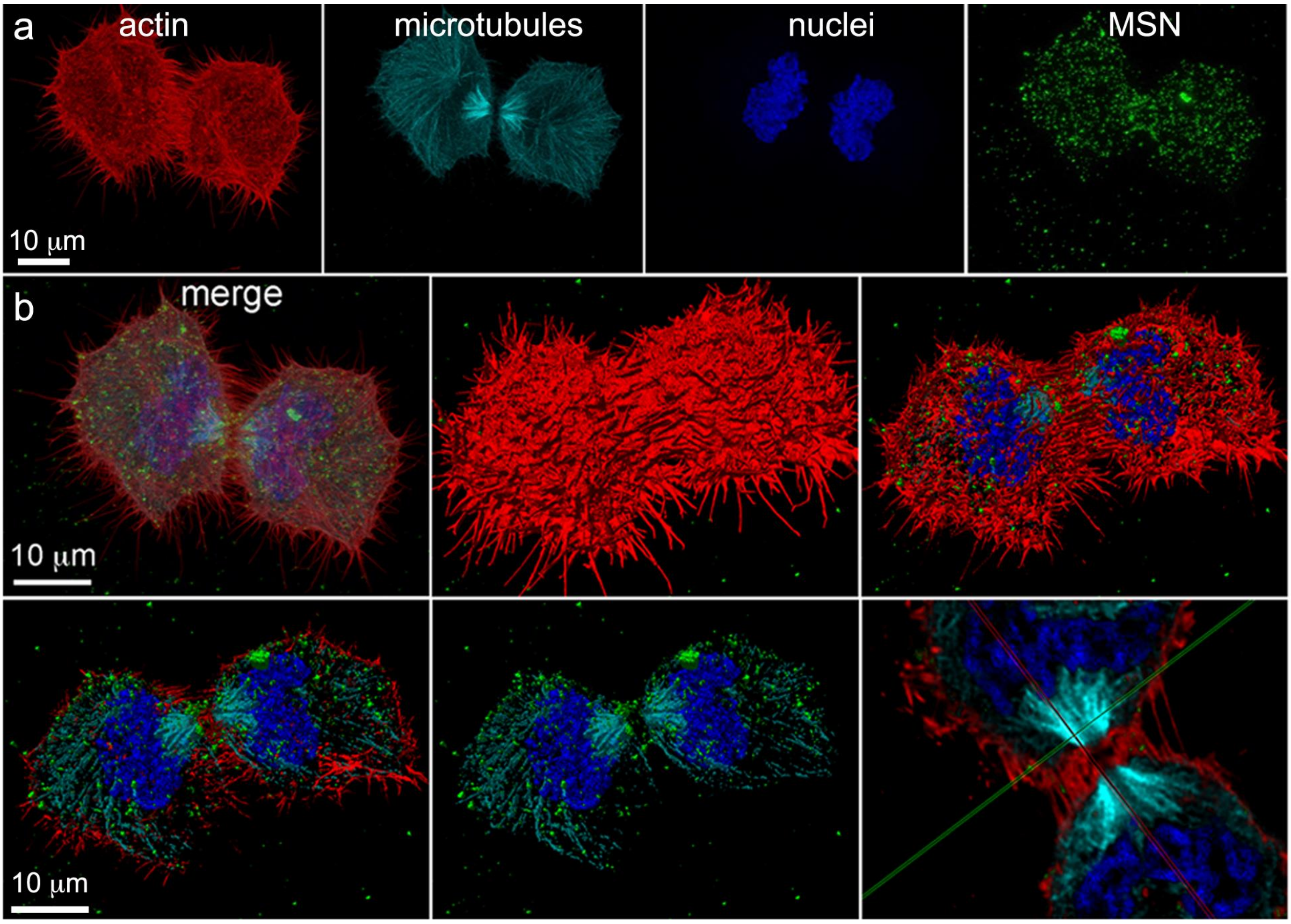

Figure S2. During late telophase-cytokinesis endosomes distribute throughout the nascent daughter cells. a-b) Confocal fluorescent micrographs of nascent daughter cells during late telophase (red: actin, phalloidin DND red; cyan: microtubules; anti-〈-tubulin antibody-AF633; green: MSN; DyLight 488; blue: nuclei, DAPI). HyVolution deconvoluted single fluorophore (a) and merged (b) fluorescent micrographs. b) 3D blend and surfacerendered images show daughter cells at two levels of actin thresholding, with sectioning of the final image shown in the bottom row.

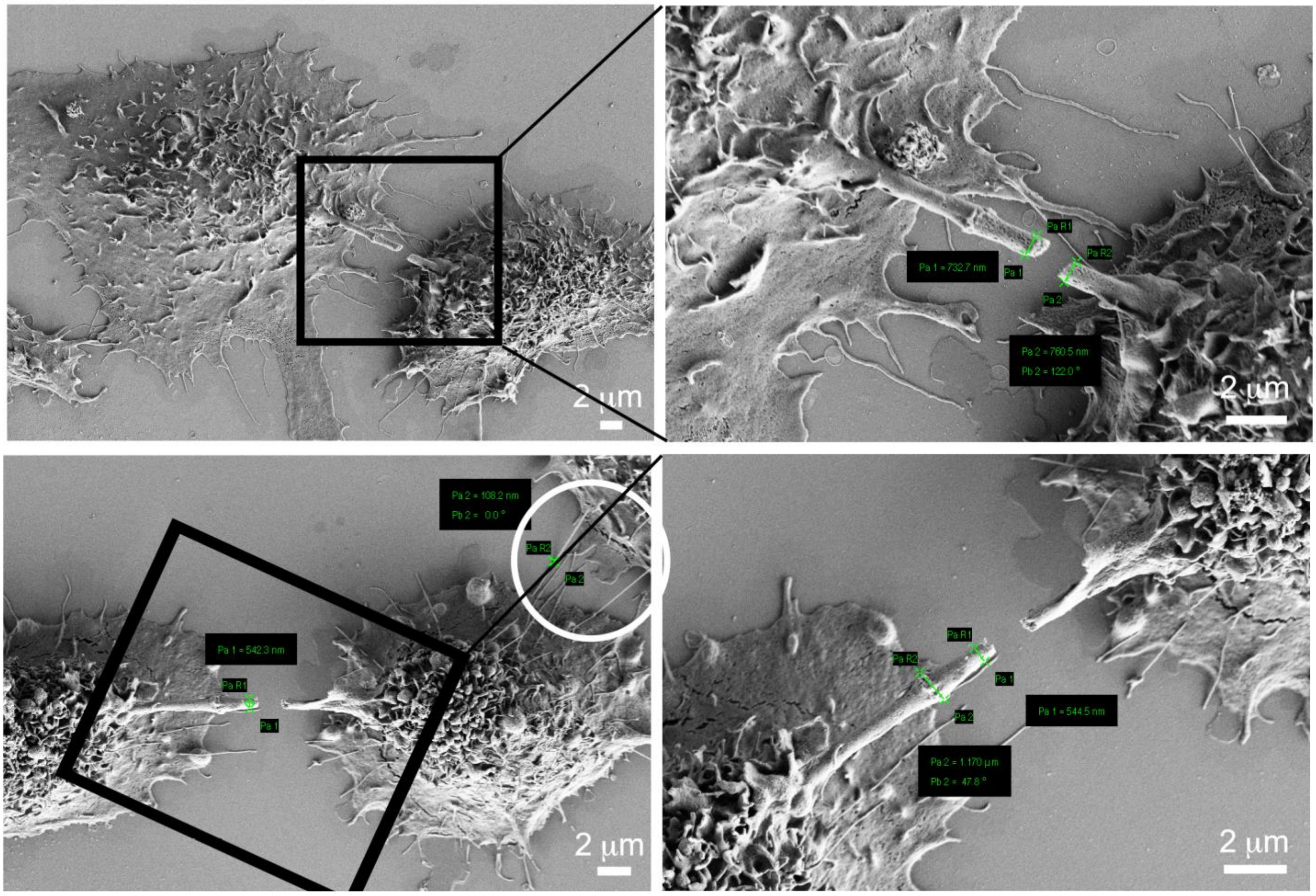

Figure S3. Mitotic nanotubes and sizing of mitotic bridges. Scanning electron micrographs show mitotic bridges connecting nascent daughter cells. The boxed regions are shown at higher magnifications. Size bars show representative diameters of mitotic bridges, a midbody, and mitotic nanotubes (shown within the white circle).

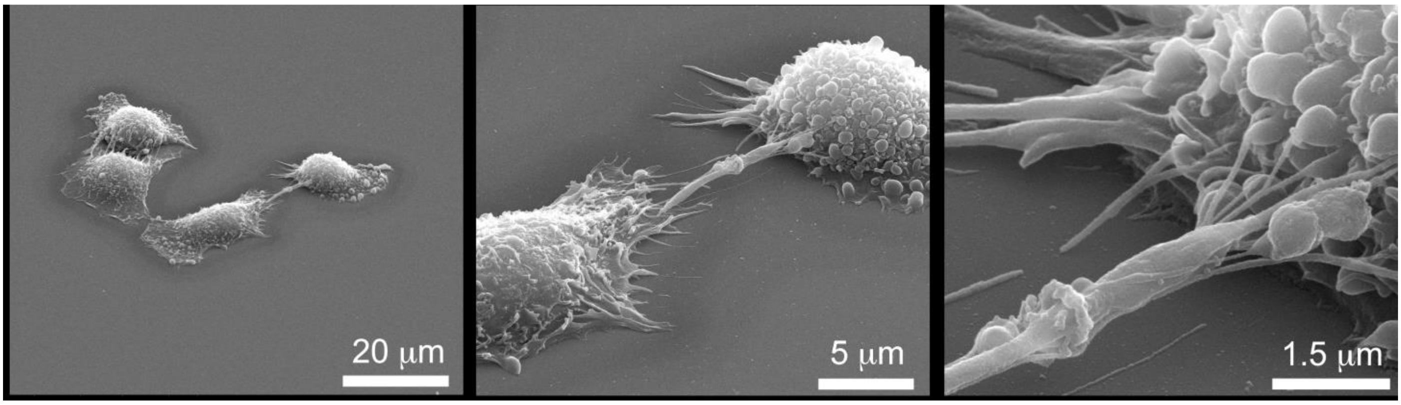

Figure S4. Topographic imaging of a mitotic bridge and midbody in A549 cells. Scanning electron micrographs showing progressive magnification of a midbody within a mitotic human A549 lung cancer cell. Images, with magnifications of 3.5k, 12k and 50k, were acquired using an FEI Quanta FEG at 20 kV.
